# Melanocortin 4 receptor agonism enhances sexual brain processing in women with hypoactive sexual desire disorder

**DOI:** 10.1172/JCI152341

**Published:** 2022-10-03

**Authors:** Layla Thurston, Tia Hunjan, Edouard G. Mills, Matthew B. Wall, Natalie Ertl, Maria Phylactou, Beatrice Muzi, Bijal Patel, Emma C. Alexander, Sofiya Suladze, Manish Modi, Pei C. Eng, Paul A. Bassett, Ali Abbara, David Goldmeier, Alexander N. Comninos, Waljit S. Dhillo

**Affiliations:** 1 Section of Endocrinology and Investigative Medicine, Imperial College London, London, United Kingdom.; 2 Invicro, a Konica Minolta Company, London, United Kingdom.; 3 Statsconsultancy Ltd., Amersham, Bucks, United Kingdom.; 4 Jane Wadsworth Sexual Function Clinic, St. Mary’s Hospital and; 5 Department of Endocrinology, Imperial College Healthcare NHS Trust, London, United Kingdom.

**Keywords:** Endocrinology, Melanocortin, Neuroimaging

## Abstract

**BACKGROUND:**

Hypoactive sexual desire disorder (HSDD) is characterized by a persistent deficiency of sexual fantasies and desire for sexual activity, causing marked distress and interpersonal difficulty. It is the most prevalent female sexual health problem globally, affecting approximately 10% of women, but has limited treatment options. Melanocortin 4 receptor (MC4R) agonists have emerged as a promising therapy for women with HSDD, through unknown mechanisms. Studying the pathways involved is crucial for our understanding of normal and abnormal sexual behavior.

**METHODS:**

Using psychometric, functional neuroimaging, and hormonal analyses, we conducted a randomized, double-blinded, placebo-controlled, crossover clinical study to assess the effects of MC4R agonism compared with placebo on sexual brain processing in 31 premenopausal heterosexual women with HSDD.

**RESULTS:**

MC4R agonism significantly increased sexual desire for up to 24 hours after administration compared with placebo. During functional neuroimaging, MC4R agonism enhanced cerebellar and supplementary motor area activity and deactivated the secondary somatosensory cortex, specifically in response to visual erotic stimuli, compared with placebo. In addition, MC4R agonism enhanced functional connectivity between the amygdala and the insula during visual erotic stimuli compared with placebo.

**CONCLUSION:**

These data suggest that MC4R agonism enhanced sexual brain processing by reducing self-consciousness, increasing sexual imagery, and sensitizing women with HSDD to erotic stimuli. These findings provide mechanistic insight into the action of MC4R agonism in sexual behavior and are relevant to the ongoing development of HSDD therapies and MC4R agonist development more widely.

**TRIAL REGISTRATION:**

ClinicalTrials.gov NCT04179734.

**FUNDING:**

This is an investigator-sponsored study funded by AMAG Pharmaceuticals Inc., the Medical Research Council (MRC) (MR/T006242/1), and the National Institute for Health Research (NIHR) (CS-2018-18-ST2-002 and RP-2014-05-001).

## Graphical abstract

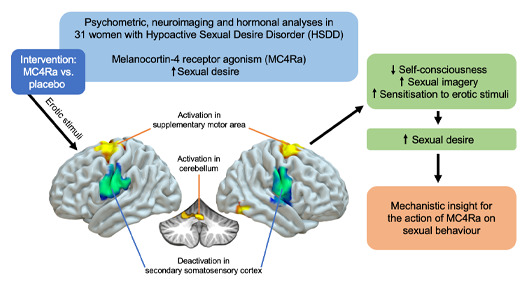

## Introduction

Sexual arousal and desire are fundamental physiological processes in humans and are designed to drive sexual activity and ultimately reproduction at a population level. The presence of sexual arousal and desire relies on complex pathways involving endocrine and neural factors (1–6). In psychosexual disorders, these physiological processes and associated pathways are frequently disrupted (7).

Hypoactive sexual desire disorder (HSDD) is characterized by a persistent or recurrent deficiency of sexual fantasies and desire for sexual activity, resulting in marked distress and interpersonal difficulties (8). The disorder is not due to a coexisting medical or psychiatric condition, problems with the relationship, or the effects of a medication or drug substance. Acquired HSDD develops in an individual who previously had no problems with sexual desire, while generalized HSDD occurs regardless of the type of stimulation, situation, or partner (7). Critically, up to 10% of women suffer from HSDD (9, 10), making it the most prevalent global female sexual health complaint. However, despite its substantial social and economic burden (11, 12), the precise underlying neural dysregulation in HSDD remains unclear. One recent meta-analysis identified a pattern of hypoactivation in lower-level, evolutionarily ancient subcortical brain regions (such as the striatum and thalamus) involved in the sexual response, with a corresponding hyperactivation in higher-level, more recently evolved cortical regions involved in self-monitoring (13). These findings suggest a neurofunctional hypothesis of HSDD, in which excessive “top-down” brain monitoring and evaluation of the sexual response may impede or prevent normal sexual functions.

There is growing evidence that the melanocortin system is involved in the neuroendocrine regulation of sexual behavior (14). Importantly, preclinical animal models suggest that the melanocortin 4 receptor (MC4R) influences sexual function, with its expression observed in a number of relevant brain regions (15–17).

Recently, a novel MC4R agonist (MC4Ra) (18) has been shown to enhance sexual desire in women with HSDD (19, 20) and has been licensed by the FDA for clinical use in premenopausal women with HSDD (21). However, the neural substrates through which MC4R agonists mediate their effects on sexual desire are currently unknown. Understanding the mechanism by which MC4R agonists mediate their effects on sexual behavior is important, not only for the ongoing development of melanocortin-based therapies for psychosexual disorders but also for obesity medicine, where related MC4R agonists are rapidly being developed (22).

**Figure 1:**
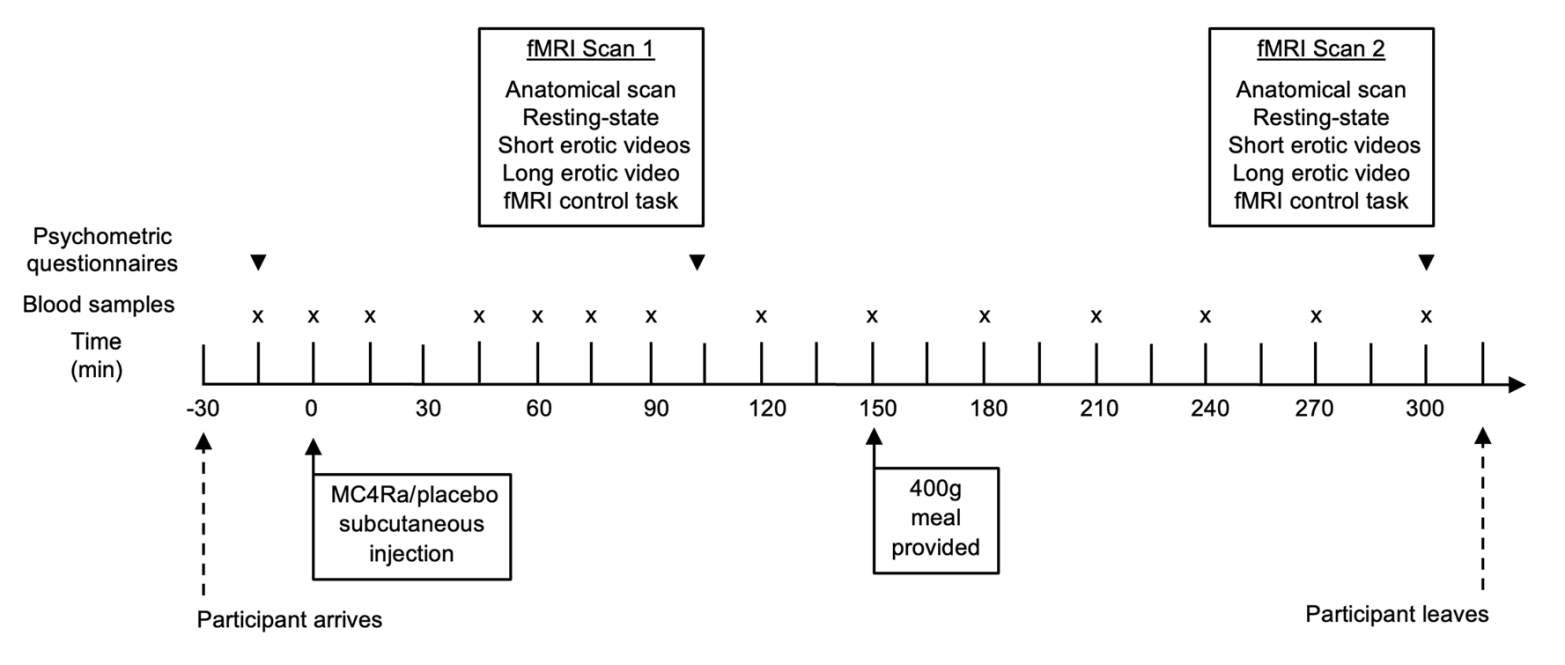
Experimental protocol. A final group of 31 premenopausal women with HSDD participated in a randomized, double-blinded, 2-way crossover, placebo-controlled study. They attended 2 study visits each: 1 for subcutaneous administration of MC4Ra and 1 for subcutaneous administration of an equivalent volume of placebo in random order. Blood samples were taken at the time points shown (denoted with an “X”). Participants underwent 2 fMRI scans and completed baseline, inter-scan, and post-scan psychometric questionnaires (denoted with an inverted triangle▼). Participants underwent two 60-minute scans per study visit, scan 1 at *t* = 45 minutes and scan 2 at *t* = 240 minutes, to cover the whole time course of possible objective and subjective responses to MC4Ra, which are known to occur from 45 minutes onward. Each 60-minute fMRI scan period included the following types of scans and tasks: anatomical and T2 proton density (to evaluate any structural abnormality and for subsequent anatomical location); resting state (to evaluate regional interactions that occur in a task-negative state, when an explicit task is not being performed); 20 × 20-second “short” erotic videos with 20 × 20-second exercise control videos, a 10-minute “long” erotic video, and an fMRI control task (to identify and control for global vascular or systemic effects of MC4R agonism).

To determine the mechanism by which MC4R agonists mediate their effects on sexual behavior, we used psychometric, functional magnetic resonance imaging (fMRI), and hormonal analyses to investigate the effects of MC4Ra on brain responses to erotic stimuli and related psychometric and hormonal parameters in women with HSDD. MC4Ra and placebo were administered on different study days to 31 premenopausal heterosexual women with HSDD (2 study visits each). We assessed the effects on sexual brain processing using psychometric, neuroimaging, and hormonal analyses in a randomized, double-blinded, placebo-controlled crossover study (Figure 1). To evaluate brain activation in the participants, we used a standard fMRI block design task, with short (20-second) videos containing erotic scenes and exercise scenes as control stimuli. Additionally, we used a “naturalistic” fMRI paradigm (23) consisting of a continuous 10-minute “long” video of erotic content to investigate changes in functional connectivity within the sexual response network in comparison with a resting-state scan as a control.

## Results

### Participant baseline characteristics.

**Figure 2:**
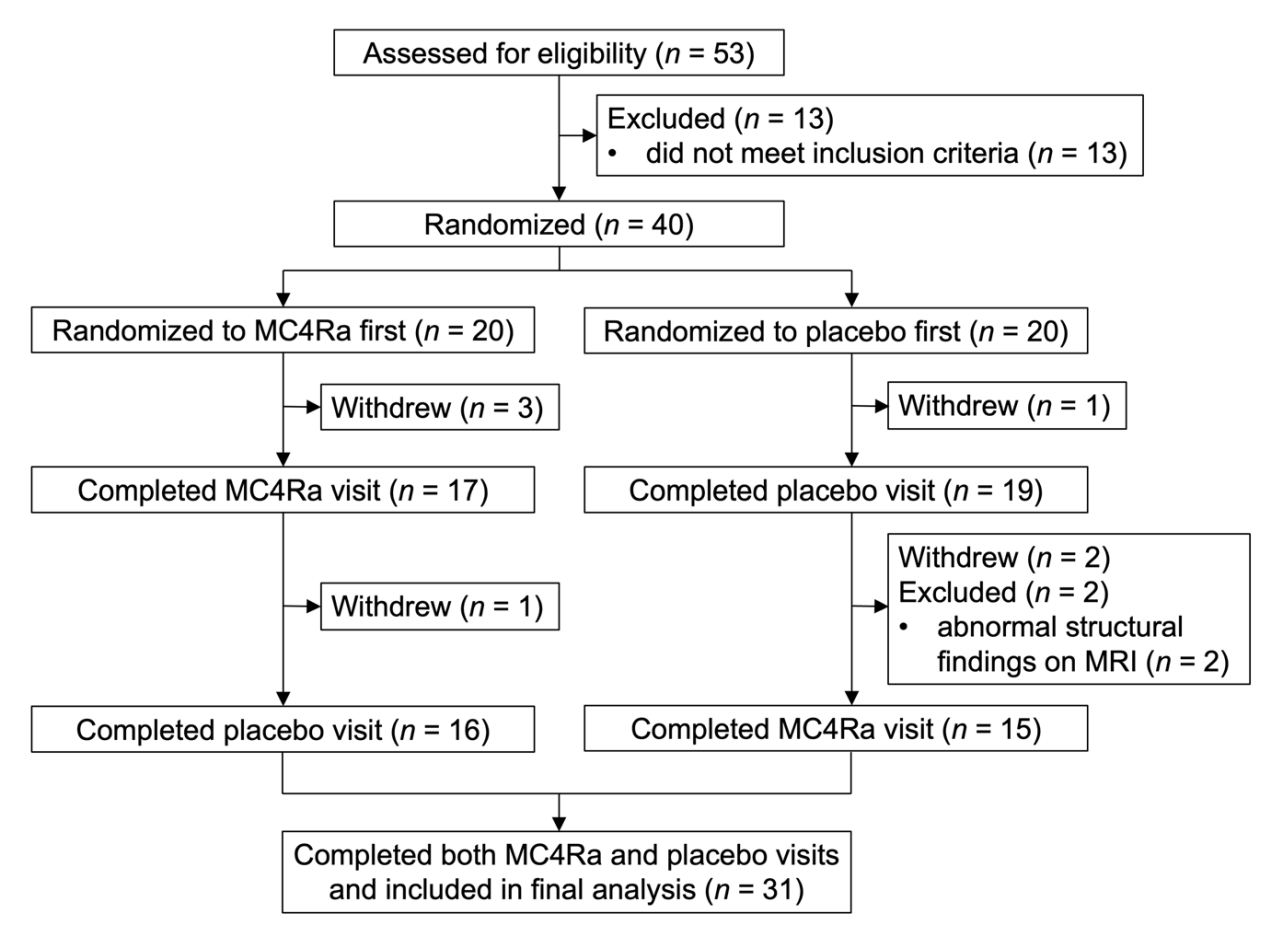
CONSORT flow diagram. Per-protocol analysis included all women with HSDD who appropriately completed both study visits, receiving both MC4Ra and placebo (final total, *n* = 31).

The CONSORT flow diagram is shown in Figure 2. The baseline characteristics of the 31 women with HSDD who completed both study visits (MC4Ra and placebo administration) are shown in Supplemental Table 1 (supplemental material available online with this article; https://doi.org/10.1172/JCI152341DS1).

### MC4Ra increased sexual desire.

**Figure 3:**
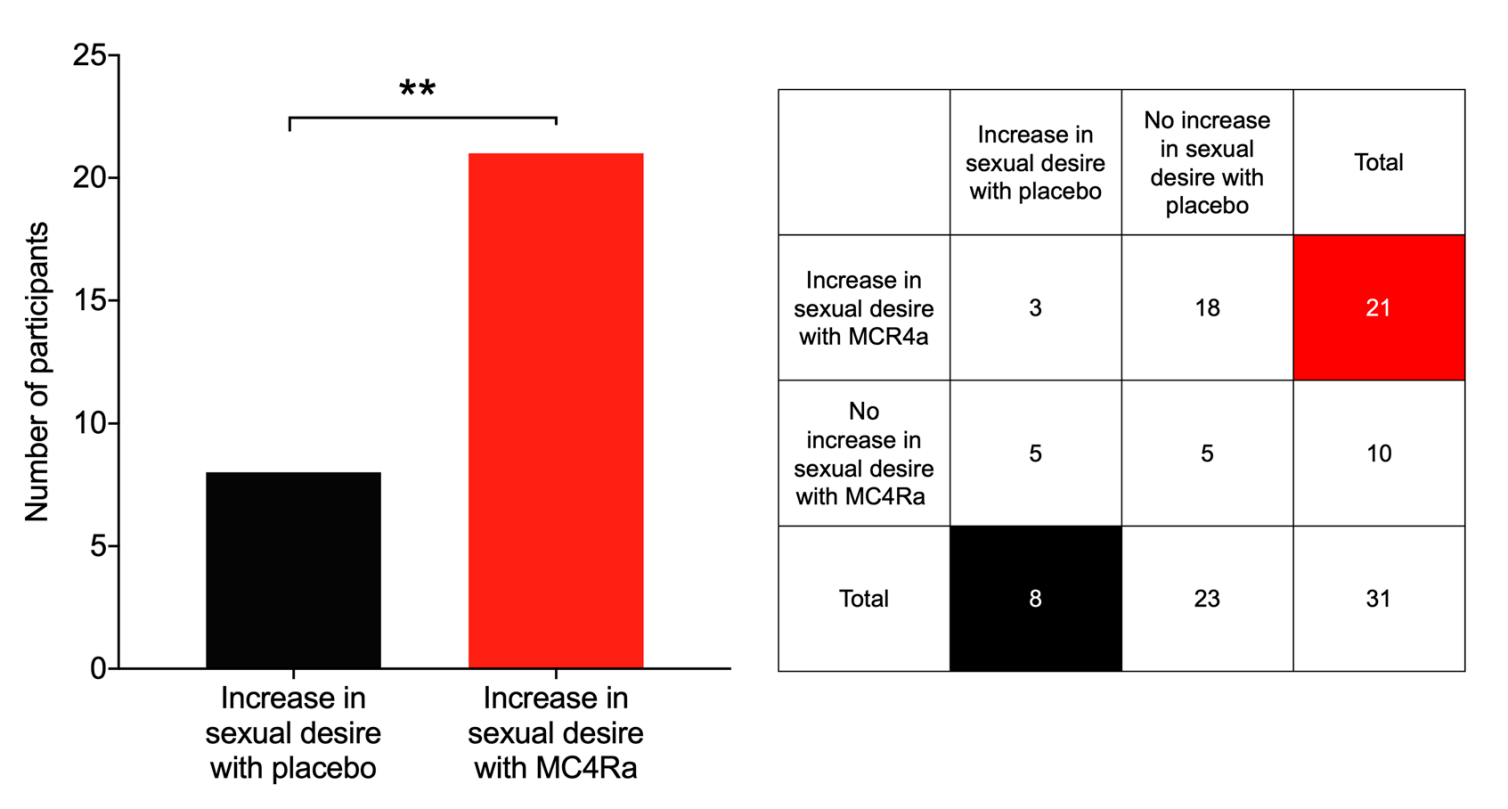
More participants reported increased sexual desire following MC4Ra administration compared with placebo. Participants were contacted 24 hours after MC4Ra or placebo administration and asked if they had experienced increased sexual desire. Data compare participants who reported an increase in desire with placebo (*n* = 8) and participants who reported an increase in desire with MC4Ra (*n* = 21). Three participants reported an increase in desire on both visits, and 5 women reported no increase in desire on either visit. A McNemar test conducted on the 2 × 2 contingency table comparing the 4 possible outcomes showed that an increase in desire on MC4Ra alone was significantly different from the other 3 outcomes **P ≤ 0.01, *n* = 31.

To provide information for the full post-administration period, participants were contacted 24 hours after each study visit and asked if they had experienced increased sexual desire in the 24 hours since administration of MC4Ra or placebo (with the investigator and participant blinded to the agent given). A significantly larger number of the participants reported increased sexual desire following MC4Ra administration compared with those who received the placebo (*P* = 0.007) (Figure 3).

### The designed tasks were effective in eliciting responses to erotic stimuli.

Averaging across both treatments and time conditions (scan 1 and scan 2) to see the general effects of the short videos task (erotic compared with exercise) identified a pattern of brain activation (see Supplemental Figure 1) consistent with that seen in previous studies using similar stimuli (24). There was strong activation in the visual cortex, cerebellum, striatum, and several dorsal sensorimotor regions. This analysis served to validate our experimental procedures by validating the tasks as effective in testing sexual brain activation.

### MC4Ra enhanced cerebellar and supplementary motor area brain activity and decreased activity in the secondary somatosensory cortex in response to erotic stimuli.

**Figure 4:**
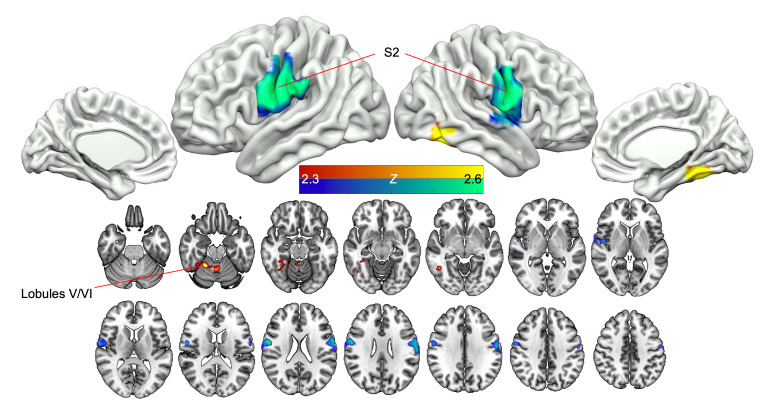
MC4R agonism enhances cerebellar activity and deactivates the S2 in response to erotic stimuli (scan 1, short video task). Red/yellow areas show relative activation in response to erotic versus exercise videos following MC4R agonist administration, compared with placebo. Blue/green shows relative deactivation in response to erotic versus exercise videos following MC4R agonist administration, compared with placebo. Significant clusters were corrected for multiple comparisons; *Z* = 2.3, *P* < 0.05, *n* = 31.

**Figure 5:**
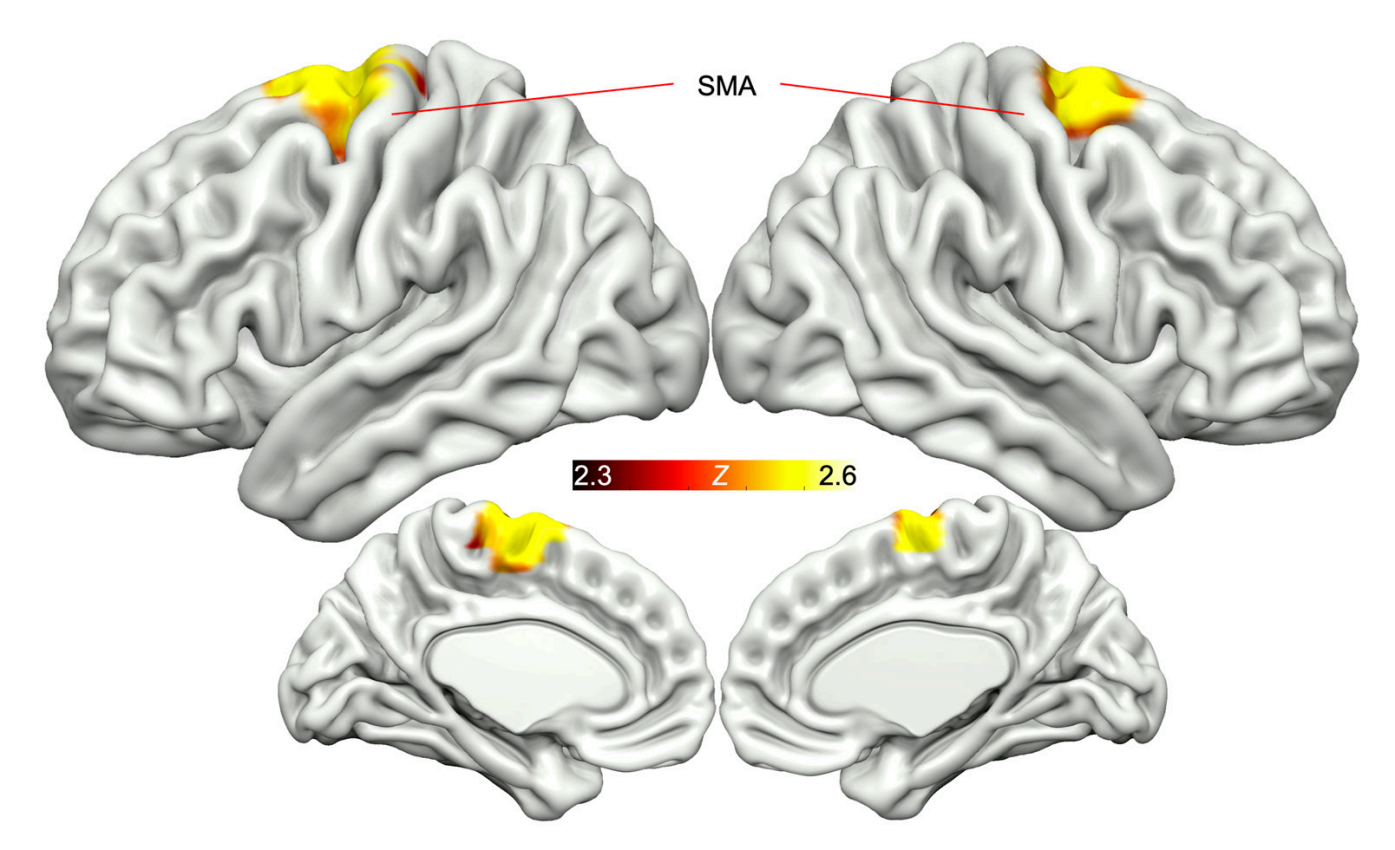
MC4R agonism activates the SMA in response to erotic stimuli (scan 2, short video task). Red/yellow areas show relative activation in response to erotic versus exercise videos following MC4R agonism, compared with placebo. Significant clusters were corrected for multiple comparisons; *Z* = 2.3, *P* < 0.05, *n* = 31.

The short video task consisted of 20-second erotic videos (depicting 1 man and 1 woman engaging in vaginal sex), alternating with neutral, nonerotic videos (depicting a man and woman engaged in exercise) as a control. In the erotic-versus-exercise (erotic-exercise) contrast during scan 1, premenopausal women with HSDD demonstrated enhanced activation in the right cerebellum (lobules V and VI) following MC4Ra administration compared with placebo. In the same contrast, we observed deactivation in the secondary somatosensory cortex (S2) bilaterally following MC4Ra administration compared with placebo (Figure 4 and Supplemental Table 2). The short video erotic-exercise contrast during scan 2 revealed enhanced activation of the supplementary motor area (SMA) following MC4Ra treatment compared with placebo (Figure 5 and Supplemental Table 2).

### The reduction of functional connectivity between the amygdala and the insula in response to erotic stimuli was prevented by MC4Ra administration.

Functional connectivity refers to the similarity between the activity of brain regions over time. Regions with similar temporal profiles are often functionally related and coordinate their activity in networks to perform particular functions (25). Previous work has also identified alterations in functional connectivity within the sexual response network in dysfunctional sexual behaviors (26).

We predefined a network of sexual function regions of interest (ROIs) that included the amygdala, the hypothalamus, the insula, the precentral gyrus, the striatum, and the thalamus. We noted significant differences in the patterns of functional connectivity within this predefined network of sexual function ROIs when the participants watched a long erotic video compared with the resting state, and between MC4Ra compared with placebo. We examined these differences using 2 (MC4Ra vs. placebo) by 2 (long erotic video vs. resting state) ANOVAs, which allowed the comparison of main effects (i.e., effects of the drug, independent of the task effects, and vice versa) and the interactions of the 2 factors (e.g., a larger difference between the 2 task conditions in 1 of the drug conditions compared with the same difference between tasks in the other drug condition). The most salient result was therefore the interaction, as it examined how the drug modulated the difference between the 2 tasks. However, interaction effects are inherently nondirectional; post hoc Tukey’s tests were therefore used to examine individual contrasts (effects of the drug within each task condition and effects of the task within each drug condition) and to assess a potential direction of effect. Separate ANOVAs were performed for each scan (1: morning; scan 2: afternoon) and for each pairing of regions in the network.

**Figure 6:**
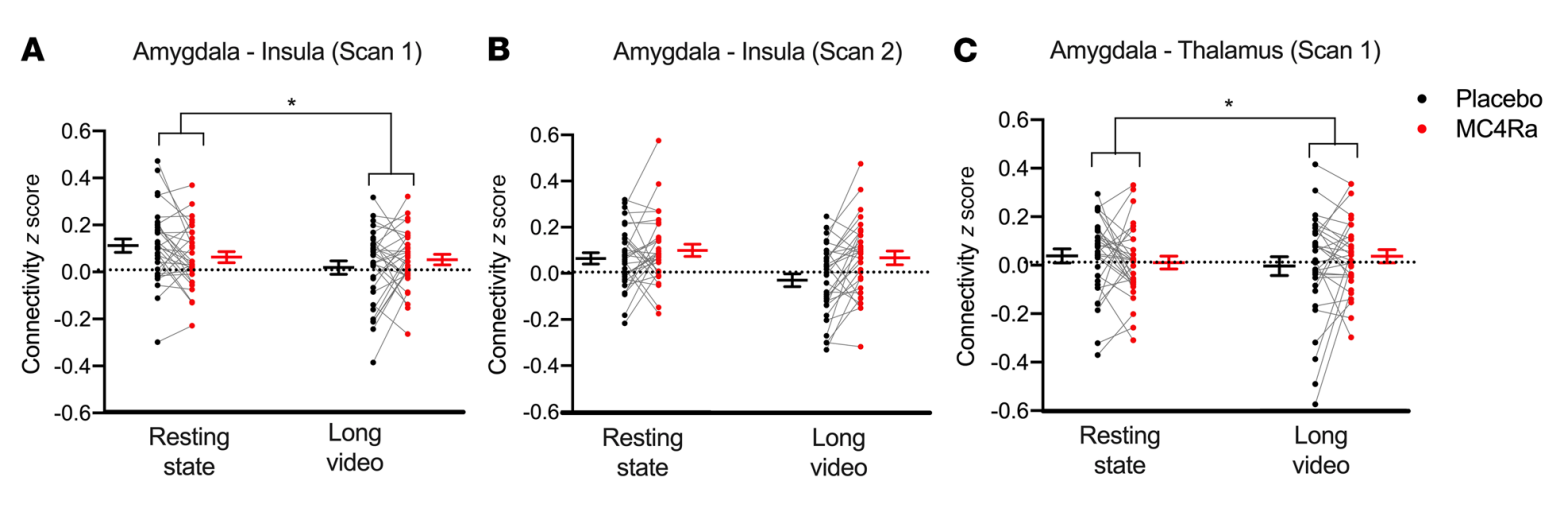
A reduction in connectivity between the amygdala and the insula in response to erotic stimuli is prevented by MC4Ra administration. Connectivity analysis of the “sexual response network” (amygdala, hypothalamus, insula, precentral gyrus, striatum, and thalamus). Panels **A**–**C** show connectivity pairings where an interaction was found by the 2 × 2 ANOVAs between the drug treatment (MC4R agonism vs. placebo) and the task (long erotic video vs. resting state). Changes in connectivity were found between the amygdala and the insula in scan 1; *F* (1,30) = 5.553, *P* = 0.025 (**A**), and scan 2; *F* (1,30) = 3.70, *P* = 0.064 (**B**), and the amygdala and the thalamus in scan 1; *F* (1,30) = 5.5043, *P* = 0.026 (**C**). Data represent the mean ± SEM. *P < 0.05, *n* = 31.

The ANOVA results from scan 1 showed a significant interaction in connectivity between the amygdala and insula ROIs (*F* [1,30] = 5.55, *P =* 0.025) (Figure 6A). This demonstrates that the difference in connectivity produced by the task (long erotic video vs. resting state) was also influenced by MC4Ra. We also noted a similar interaction trend for this pairing in scan 2 (*F* [1,30] = 3.70, *P* = 0.064) (Figure 6B). A significant interaction effect was also seen in the amygdala-thalamus pairing in scan 1 (*F* [1,30] = 5.50, *P* = 0.026) (Figure 6C). No further interactions were observed in other pairings in either scan.

The interaction effects seen in Figure 6, A–C, appear to have been driven by the placebo condition, in which there was a reduction in the connectivity during the long video compared with that seen in the resting state. Conversely, connectivity remained relatively unchanged in the MC4Ra condition.

To investigate this further, we performed post hoc Tukey’s tests. In the amygdala-insula pairing in scan 1 (Figure 6A), the post hoc Tukey’s tests revealed that the resting state and long video scans were significantly different in the placebo condition (*t* [30] = 3.157, *P* = 0.018). In the amygdala-insula pairing in scan 2, the post hoc Tukey’s tests showed a statistically significant difference between the placebo resting state and long video scans (*t* [30] = 4.656, *P* < 0.001). There was also a difference in the connectivity between the long video scans, where connectivity was higher in participants while on MC4Ra compared with placebo (*t* [30] = 3.473, *P* = 0.008). In addition, there was a significant difference between the resting-state MC4Ra scan and the long video placebo scan (*t* [30] = 3.938, *P* = 0.002). We observed no other significant effects in the post hoc tests.

Taken together, these data suggest that there was a reduction of functional connectivity between the amygdala and the insula in response to erotic stimuli and that this reduced connectivity was prevented by MC4Ra administration.

### MC4Ra had no confounding effects in the fMRI control task.

The purpose of the control task (auditory, motor, and visual stimuli) was to detect any systemic effects (e.g., on cerebral blood flow) of the study drug, which may have nonspecific effects on the blood oxygen level–dependent (BOLD) response and may therefore confound the results from the erotic tasks. We observed no effects of the study drug on this task in any of the conditions, confirming that there were no confounding effects of MC4Ra on the BOLD response (see Supplemental Figure 2) and thus adding further validity to our observed results.

### MC4Ra resulted in a small increase in circulating luteinizing hormone, follicle-stimulating hormone, and testosterone.

MC4Ra administration resulted in a small mean increase in luteinizing hormone (LH) of 1.1 iU/L (*F* [1,58] = 13.38, *P* = 0.0005), follicle-stimulating hormone (FSH) of 0.35 iU/L (*F* [1,60] = 10.97, *P* = 0.0016), and testosterone of 0.09 nmol/L (*F* [1, 60] = 4.213, *P* = 0.005) over the 300-minute study duration (Supplemental Figure 3). We observed no effect on circulating estradiol or progesterone levels (Supplemental Figure 3).

### MC4Ra increased satiety and reduced food intake.

MC4R agonism is well known to cause nausea (27) and reduced appetite (28). As expected, MC4Ra treatment led to an increase in nausea (after scan 1 and scan 2) and an increase in the feeling of fullness (after scan 1) (Supplemental Figure 4, A–C). Objectively, food intake was reduced after scan 1 (Supplemental Figure 4D).

### MC4Ra had no effect on attention.

Attention was not affected by MC4Ra (Supplemental Figure 4E), thus excluding a further possible additional confounder for the observed brain effects in our study.

## Discussion

This is the first study to our knowledge to investigate the effects of MC4R agonism on sexual brain processing in women with HSDD and reveals several notable findings from the multimethod approach we used. First, an increase in sexual desire was reported following MC4Ra administration. Second, MC4Ra elicited significant (*P* < 0.05) effects on the brain response to erotic stimuli, both in terms of patterns of focal relative activation and deactivation (short videos task) and in connectivity between areas established in sexual function (long videos task). Third, MC4Ra caused small increases in the levels of circulating LH, FSH, and testosterone, with no effect on estradiol or progesterone levels.

The female sexual response is dependent on physiological, psychological, and social factors. Key regions in the brain that form the sexual desire brain network (SDBN) (13) include the prefrontal cortex, the locus coeruleus, the medial preoptic area, the paraventricular nucleus, and reward- and attention-processing centers of the ventral tegmental area and the nucleus accumbens.

The presumed pathogenesis of HSDD pertains to a dysregulation of the following neural pathways: central sexual excitatory (dopamine, noradrenaline, melanocortin, and oxytocin) and sexual inhibitory (serotonin, opioid, endocannabinoid, and prolactin) networks in the prefrontal cortex and limbic system (29, 30). Moreover, a meta-analysis of female sexual desire and HSDD neuroimaging studies proposed that HSDD is associated with a specific fronto-limbic-parietal dysfunction characterized by reduced activation of the SDBN with increased activation of the self-referential brain network (SRBN) (13). The SRBN includes brain areas involved in self-referential functions. For example, areas such as the medial prefrontal cortex, the supramarginal gyrus, and the inferior parietal lobule are believed to be involved in self-focus, spectatoring (focusing on oneself from a third-person perspective), and egocentrism. The putamen and precentral gyrus are implicated in shyness and moral judgment (31, 32), and the visual cortex and fusiform gyrus are believed to be involved in visual analyses and processing bodies and faces (33). It is hypothesized that self-monitoring of the sexual response in these women interferes with the processing of erotic stimuli (13).

Sexual therapy and education presently form the basis of treatment for HSDD, with limited pharmacologic therapeutic options available (34). Flibanserin is a 5-HT_1A_ agonist/5-HT_2A_ antagonist that is licensed by the FDA for the treatment of premenopausal women with HSDD. In addition to requiring daily administration, this drug has only a modest treatment effect, accompanied by significant side effects (such as dizziness, somnolence, and nausea) and has a marked adverse interaction with alcohol (35). Transdermal testosterone therapy is licensed in the United Kingdom but only for use in postmenopausal women (36). The MC4Ra bremelanotide, an “as required” subcutaneous injection, was approved by the FDA in 2019 for premenopausal women with generalized, acquired HSDD. However, the mechanism by which MC4Ra mediates effects on sexual behavior is unknown. We thus sought to define the brain processes underpinning MC4Ra effects in this regard.

In this study, MC4R agonism caused a significant (*P* ≤ 0.01) increase in participant-reported sexual desire compared with placebo up to 24 hours after administration. This finding is consistent with the phase III trials of bremelanotide, in which participants reported an increase in desire according to the female sexual function index (FSFI) questionnaire (20). The mechanistic pathway behind this increase in sexual desire may be explained by the observed changes in specific erotica-induced brain activity observed on fMRI in the current study.

In the short video erotic compared with exercise contrast, MC4Ra deactivated a distinct bilateral region in the parietal operculum, with the posterior edge of the cluster extending back into the temporo-parietal junction and the supramarginal gyrus. The most plausible functional designation of this region is the S2. Modern conceptions of the role of S2 emphasize its role in high-level somatosensory functions, meaning it integrates somatosensory information with social, emotional, and other information in order to generate a holistic sense of our bodily representation in the world (37). Other closely related regions around the temporo-parietal junction have also been implicated in interoceptive processes and functions related to body ownership (38). A meta-analysis of HSDD brain activation studies (13) also identified small clusters in these regions as being hyperactive in women with HSDD. The relative deactivation of S2 by MC4R agonism observed in the current study may therefore enable women with HSDD to relax their higher-level control and self-monitoring of the sexual response and provides a neural mechanism for an increase in sexual desire in response to erotic stimuli. This result is consistent with the “top-down” inhibition theory of HSDD (13), whereby cognitive interference inhibits visceral/limbic/somatosensory stimulation pathways (39).

During scan 1, MC4Ra increased activation of the right cerebellum during the short erotic videos. Specifically, this was observed in the right hemisphere lobules V and VI, which are specialized for sensorimotor functions (40). Cerebellar activation is related to the “feeling” experience associated with sexual arousal (41), and a meta-analysis of neuroimaging studies reported cerebellar activation with sexual arousal in 14 studies (42). Furthermore, cerebellar activation was found to be significantly blunted in women with HSDD (43) and conversely increased in healthy participants upon viewing erotic videos (44). Previous studies have also described cerebellar activation during the female orgasm (45–47); in addition, the right cerebellum has been shown to correlate with changes in genital temperature (48). Interestingly, rodent studies have identified MC4Rs within the cerebellum (17, 49). Therefore, the increased activity in lobules V and VI observed following MC4Ra administration in this study is consistent with the general pattern of modulation of the brain’s somatosensory system seen in our cortical results.

During scan 2, MC4Ra increased activation in a dorso-medial region, centered at the junction of the precentral and superior frontal gyri. Functionally, this region is designated as the SMA, and its role in motor functions is analogous to the S2’s role in somatosensory information, that of high-level integration of motor operations with other domains. The SMA has been reported to respond to visual erotic stimuli (50) and has been identified as part of the cognitive component of the visually induced sexual arousal response model (42, 51, 52). The cognitive component includes a stimulus appraisal process allowing categorization and evaluation of erotic stimuli. It also confers increased attention to visual stimuli that are categorized as sexual. In addition, sexual motor imagery is mediated in part by the SMA (53, 54). Sexual motor imagery is defined as a dynamic state, whereby a motor act is internally rehearsed within one’s memory without overt actions, occurring when an individual observes an action and wishes to imitate it (55). Indeed, it has previously been demonstrated that there is greater activation of the SMA in response to visual erotic stimuli in healthy individuals when compared with patients with HSDD, indicating a lack of sexual motor imagery in these patients (54). In line with these findings, our study showed that MC4Ra increased SMA activation, providing evidence for this mechanism of increased sexual motor imagery.

In functional connectivity analyses using the long video (a 10-minute erotic video) and resting-state scans (as a control), we found that MC4Ra increased functional connectivity between the amygdala and the insula, as well as between the amygdala and the thalamus. These 3 nodes are important regions in the normal response to erotic stimuli, are key parts of the brain’s limbic system, and are involved in a range of other lower-level processes (such are disgust, threat aversion, facial emotion perception) with fundamental biological relevance. The pattern of results in these analyses suggests that functional connectivity under placebo conditions was relatively high in the resting state but was decreased by exposure to the erotic stimulus in women with HSDD. This may be because when women with HSDD are exposed to erotic stimuli, they interpret this as a negative rather than a positive stimulus, with this suppression of connectivity due to the hyperfunctional “top-down” inhibition of sexual desire pathways (13). Interestingly, we found that MC4Ra administration prevented this reduction in connectivity in women with HSDD, even while viewing the erotic stimulus, suggesting either that the lower-level processes were relatively more stimulated or that the “top-down” suppression had been mitigated. This pattern of results also fits the neurofunctional theory of HSDD (13) and thus provides further mechanistic insight into MC4R agonism.

With respect to reproductive hormone levels, MC4Ra administration resulted in a small increase in circulating LH, FSH, and testosterone levels compared with placebo. Previous studies in rodents (56, 57) and humans (58, 59) have described a role for α-melanocyte–stimulating hormone (α-MSH), which binds to the MC4R, in the regulation of the hypothalamic/pituitary/gonadal (HPG) axis. Furthermore, melanocortin neurons are modulated by estradiol and play an important role in the negative feedback of the HPG axis, and α-MSH activation of the MC4R increases gonadotrophin-releasing hormone (GnRH) action potential firing (60). It is unlikely that the behavioral and brain effects we observed were due to these LH and FSH changes, as they were small, and LH and FSH are not known to have roles in sexual behavior in humans. With regard to testosterone, studies testing transdermal testosterone therapy for low libido in premenopausal women have demonstrated an improvement in sexual function associated with a mean serum testosterone increase of 0.52–1.54 nmol/L (36, 61, 62), although other large cross-sectional studies have not found a relationship between testosterone and HSDD or sexual desire (36, 63). Given that the mean change in testosterone levels was significantly smaller (0.09 nmol/L) than what was seen in the aforementioned interventional studies, it is unlikely that this change significantly contributed to the behavioral or neural effects we observed but should be noted.

This is the first study to our knowledge to examine functional brain connectivity in women with HSDD, an area previously highlighted as requiring further investigation in these patients (13). The strengths of this study are that it was appropriately powered with several methodologies applied and was controlled for menstrual cycle and hormonal contraceptive use. The fMRI tasks were robust, as evidenced by group means and uniform task lengths and were carefully controlled for, using exercise videos with an a priori analysis plan. In addition, erotic stimuli were in the form of videos, which are known to elicit more robust responses than do pictures alone (64). Furthermore, the fMRI control task controlled for any pharmacological vascular effect, and fMRI data analysis examined both brain activation and connectivity. All participants acted as their own controls and interacted with the same female doctor throughout, thereby minimizing variability in instructions given between participants and reducing bias associated with mixing investigators of different sexes (65). Study limitations include variations in potential subjective arousal from the erotic stimuli, although using an independent focus group to rate and select the videos minimized this. Also, viewing erotic stimuli in a scanner may have the potential to affect ecological validity, however, similar methods have previously been used in robust studies of brain activation patterns in women with HSDD (43, 44, 66). While the findings of this study are applicable to premenopausal women with HSDD, it would be interesting to extend the study to postmenopausal women and men with HSDD. In addition, given that HSDD occurs in individuals of all sexual orientations (67), it would be important in future studies to explore brain responses to MC4Ra in individuals with HSDD of sexual orientations other than heterosexual. Furthermore, it would be interesting to assess brain responses to different-orientation (to the participant) erotic material to explore the condition further. Finally, studies of MC4R agonists have demonstrated ongoing biological effect with repeated use (20, 68), therefore, it would be instructive to perform a longitudinal study of repeated MC4Ra administration to explore the longer-term effects on brain processing.

MC4Ra-based therapies have thus far been successfully developed principally as treatments for obesity (22). As such, we believe this study provides important clinical relevance in this regard as, for the first time to our knowledge, we have demonstrated human fMRI brain changes following subcutaneous administration of this type of compound. Indeed, alterations in erectile function have been reported with the recently licensed MC4Ra setmelanotide (22).

In summary, these data define the neural substrates and connections through which MC4R agonism modulates sexual brain processing to increase sexual desire. These changes in brain activation reduce self-monitoring and spectatoring of the sexual response, increase sexual imagery, and sensitize women with HSDD to erotic stimuli. We therefore provide insight into the mechanism by which MC4R agonism affects sexual behavior in women with HSDD, with important implications for the future development of safe and effective treatment options for women with HSDD as well as the broader use of MC4R agonists.

## Methods

### Participants.

Heterosexual premenopausal women concerned and/or distressed by low sexual desire and who had regular monthly menstrual cycles were invited to take part in this study via advertisements. Potential participants were initially telephone screened and subsequently underwent a detailed medical examination screening visit. Blood tests were performed during the screening visit to confirm health status and measured the following: complete blood count, renal function, liver function, bone profile, thyroid hormone profile, and levels of LH, FSH, estradiol, progesterone, testosterone, sex hormone–binding globulin (SHBG), dehydroepiandrosterone sulphate (DHEAS), and androstenedione. Reproductive hormone levels were consistent with premenopausal status (Supplemental Table 1).

Inclusion in this study required a diagnosis of generalized, acquired HSDD of at least 6 months’ duration as per the criteria of the Diagnostic and Statistical Manual of Mental Disorders, 4th Edition, Text Revision (DSM-IV-TR) (8), confirmed with a FSFI score of score of 26 or lower and a score of 5 or lower in the desire domain (69), as well as a score of 18 or higher on the Female Sexual Distress Scale – Desire/Arousal/Orgasm (FSDS-DAO) assessment tool (70). Participants completed the Patient Health Questionnaire-9 (PHQ-9) and the General Anxiety Disorder-7 (GAD-7) questionnaire to exclude depression and anxiety, respectively.

Other inclusion criteria were: right handedness, involvement in a stable and monogamous relationship for at least 6 months, no use of any form of hormonal contraception, absence of current or past psychiatric illness, no use of psychoactive substances (prescribed or illicit) for a minimum of 6 months prior to screening, a BMI of 18–35 kg/m^2^, and normal or corrected-to-normal vision. Exclusion criteria were: pregnancy, breastfeeding, a history of unresolved sexual trauma or abuse, and a contraindication to MRI scanning.

### Sample size.

To our knowledge, there are no previous fMRI studies examining the role of the MC4R in women with HSDD. However, data from a previous fMRI study (71) examining a similar scenario (hormonal administration effects on fMRI sexual brain activity) were used to estimate requirements for the current study. This study showed that a behavioral hormone enhances BOLD signal change in the limbic structures by a mean of 0.74% and a SD of 0.38% compared with vehicle (mean, 0.48%; SD, 0.51%). In anticipation of a similar response in this study, with a 5% significance level and 80% power, and assuming a correlation between MC4Ra and placebo results of 0.4, the sample size of this study included 31 participants. To allow for natural variation in responses, dropout, and exclusion of 20%, 40 participants were recruited to the study. In addition, this sample size is in keeping with empirically derived estimates to allow sufficient power to detect moderate-sized effects in fMRI studies (72), as well as noninterventional fMRI studies in women with HSDD (66), and our previous work examining the hormonal effects of kisspeptin versus vehicle on brain activity in healthy volunteers (71, 73–79). Following screening and informed consent, 40 participants were randomized to take part, with 31 participants completing both MC4Ra and placebo study visits (Figure 2).

### MC4Ra and placebo.

The MC4Ra used in this study was bremelanotide, manufactured by AMAG Pharmaceuticals Inc. Bremelanotide 1.75 mg/0.3 mL for subcutaneous administration was an aqueous formulation that consisted of bremelanotide 1.75 mg and 2.5% (weight per volume), glycerin (multicompendial vegetable grade, United States Pharmacopeia) in sterile water for injection, with either hydrochloric acid or sodium hydroxide (National Formulary) to adjust for pH. Placebo 1.75 mg was an equivalent prefilled autoinjector without the active ingredient in an equivalent 0.3 mL solution volume. Both products were labeled and packed in full compliance with good manufacturing practice (GMP) requirements. Subcutaneous autoinjector pens were stored below 25°C.

### Study design.

We performed a randomized, double-blinded, 2-way crossover, placebo-controlled clinical study. A total of 31 participants completed the study (Figure 1). On 1 visit, the participants received the MC4Ra, and on the other visit, they received the placebo, which was packaged in materials identical to the MC4Ra packaging, as described above. Studies were scheduled at least 1 month apart (mean, 1.32; SEM, 0.12) to ensure full washout between visits and to allow the study to be performed at the same stage of the participant’s menstrual cycle each time. Sixteen women received MC4Ra on their first visit, and 15 received placebo. The participants acted as their own controls, thereby minimizing the effects of interparticipant variation and maximizing the power of the study.

All studies were undertaken on days 1 to 7 of the menstrual cycle (follicular phase) to ensure consistent reproductive hormone levels, as brain activity can be altered by significant fluctuations in reproductive hormones across the menstrual cycle (80). The participants were asked to abstain from sexual activity, alcohol, caffeine, and tobacco from midnight prior to each study visit and were asked to have their normal breakfast on the study days. Figure 1 illustrates the study protocol.

An intravenous cannula was inserted into the arm for blood collection at 15- to 30-minute intervals. Participants completed the psychometric questionnaires as detailed below. At *t* = 0 minutes, MC4Ra or placebo (identical in volume and appearance), in the form of a single-use autoinjector, was administered subcutaneously into the abdomen. Participants and data analysts were blinded to the injection identity, the order of which was randomized in a balanced manner by an independent statistician.

### Assays.

Blood samples were collected at the time points indicated in Figure 1. Serum levels of LH, FSH, estradiol, progesterone, and testosterone were measured using automated chemiluminescence immunoassays (Abbott Diagnostics). Interassay coefficients of variation were as follows: LH, 3.4%; FSH, 3.5%; estradiol, 3.4%; progesterone, 1.8%, and testosterone 4.6%. The limits of detectability for each assay were as follows: LH, 0.07 IU/L; FSH, 0.05 IU/L; estradiol, 70 pmol/l (19 pg/mL); progesterone, 0.3 nmol/L (0.1 ng/mL); and testosterone, 0.08 nmol/L.

### Psychometric questionnaires.

On arrival, the participants were asked to complete the Sexual Arousal and Desire and Inventory (SADI) to assess multidimensional sexual arousal and desire (81). There were no differences observed between groups in any domain of the SADI questionnaire (Supplemental Figure 5). Participants were also asked to rate their satiety and nausea levels using a visual analog scale (82) in order to co-assess the established effects of MC4R agonism on appetite and nausea (20, 22). These questionnaires were repeated following both fMRI scans. Attention, a possible confounder, was assessed using the D2 Test of Attention, performed at the end of scan 2 (83). Lunchtime food intake was measured by weighing the remaining food after a participant-selected 400 g meal. Twenty-four hours after MC4Ra or placebo administration, the participants completed a follow-up questionnaire, in which they were asked to report any change in sexual desire.

### MRI procedure.

Participants underwent 2 scans per day — scan 1 at *t* = 45 and scan 2 at *t* = 240 minutes — to cover the complete time course of possible objective and subjective responses following MC4Ra administration, which are known to occur from 45 minutes onward. The fMRI scans lasted 60 minutes. Each fMRI scan session included the following types of scans and tasks: anatomical and T2 proton density (to evaluate any structural abnormality and for subsequent anatomical location); resting state (to evaluate regional interactions that occur in a task-negative state, when an explicit task is not being performed); 20 × 20-second “short” erotic videos with 20 × 20-second exercise control videos; a 10-minute “long” erotic video; and an fMRI control task (to identify and control for global vascular or systemic effects of MC4R agonism).

A mirror mounted on the head coil allowed participants to view a screen mounted in the rear of the scanner bore, where visual stimuli were back-projected through a wave guide in the rear wall of the scanner room. Participants also wore headphones to receive instructions and associated auditory stimuli during the clips. For safety monitoring, a pulse-oximeter was attached to the participant and connected to a standard data-recording system (AD Instruments PowerLab) in the control room.

### Short erotic videos task.

Erotic stimuli consisted of 20-second erotic videos alternating with neutral nonerotic videos as a control, in a standard validated block design. During the scans, participants were asked to rate their subjective level of arousal on a 5-point scale using a 5-button hand-held device after each video, with no difference observed between MC4Ra and placebo visits. The rating period lasted 5 seconds and was followed by a 10-second blank gray screen, which provided a baseline/resting condition. The erotic videos were the top-20-rated videos (of 80 videos) for sexual arousal by an independent focus group comprising 20 healthy heterosexual women. All videos contained 1 woman and 1 man engaging in vaginal sex (erotic videos) or performing exercises (control videos). The task lasted a total of 12 minutes.

### Long erotic videos task.

Participants were shown a 10-minute erotic video and asked to rate their subjective level of arousal in real time using an MR-compatible scroll wheel (behavioral potentiometer) to ensure attention. The video was sized to take up approximately 90% of the viewing area on the screen, with the bottom 10% of the screen containing a scale running from “Not at all sexually aroused” (far left) to “Very sexually aroused” (far right). The scroll wheel controlled a triangular marker that the patient could also see on the screen, and they could move the marker along the scale (left or right) as and when they desired throughout the erotic video. Participants were shown a different video in scan 1 and scan 2. No difference was observed between MC4Ra and placebo visits. The videos were the 2 highest rated (of 20 possible videos) for sexual arousal by an independent focus group comprising 5 healthy heterosexual women. The total task time was 10 minutes, plus a 10-second buffer period at the end (blank gray screen) to ensure capture of the latter portion of any brain response.

### fMRI control task.

The fMRI control task was designed to control for the potential issue in pharmacological fMRI studies, in which a drug may have confounding effects on physiological processes (e.g., cerebral blood flow) that can affect the BOLD response independently of any neural effects of the drug (84). This was the same as the validated task described by Harvey et al. (85), which was a fast event–related design consisting of 20 each of visual, auditory, motor, and eye-movement trials, plus an additional 20 null trials, to give 100 trials in total. Each trial lasted 3 seconds, to give a total task time of 300 seconds (5 minutes), plus a 10-second buffer period at the end. A small, square red fixation point was present throughout the task. On visual trials, a sinusoidal visual grating was displayed that drifted rapidly left to right and reversed direction every 0.5 seconds. On auditory trials, a sequence of 6 pure tones was presented through the headphones at different pitches, in random order. On motor trials, a blue button appeared on the screen 3 times, and participants were asked to respond with a button-press each time. On eye movement trials, the fixation point moved around the screen to random locations every 0.5 seconds, and the participants were asked to follow it with their eyes. These events were presented in a pseudo-randomized sequence, with different sequences used for scan 1 and scan 2.

### fMRI data analysis.

fMRI data processing was performed using FEAT (fMRI Expert Analysis Tool), part of the Oxford Centre for Functional MRI of the Brain (FMRIB) Software Library (FSL), version 6.0 (www.fmrib.ox.ac.uk/fsl). Registration to high-resolution structural image was carried out using the FMRIB Linear Image Registration Tool (FLIRT) (86). Registration from the high-resolution T1 structural image of each participant to the standard Montreal Neurological Institute (MNI) 152 space was then further refined using FMRIB’s Nonlinear Image Registration Tool (FNIRT) (87, 88). The following prestatistical processing was applied: motion-corrected FLIRT (86), non-brain removal using the Brain Extraction Tool (BET) (89), spatial smoothing (6.0 mm), and high-pass temporal filtering (90 seconds for the short videos, 100 seconds for the long videos and resting state). All first-level models included the extended set of head motion parameters regressor (original parameters, plus derived temporal derivatives and quadratic functions). White matter and cerebral spinal fluid masks were created from each participant’s anatomical scans using the FMRIB’s Automated Segmentation Tool (FAST), and the time series from each functional scan was extracted from these masks for use as a regressor of no interest for each participant in each task to further denoise the data. Time-series statistical analysis was carried out using FMRIB’s Improved Linear Model (FILM) with local autocorrelation correction (90).

### Short videos and control task.

The regressors of interest were derived from the onset times of the stimulus conditions and were convolved with a gamma function to simulate the hemodynamic response function (HRF). These were used as the main regressors of interest in the general linear model (GLM) with the denoising methods mentioned above as regressors of no interest. The contrasts were defined by each stimulus condition compared with baseline, followed by comparison of 2 stimulus conditions of interest, with contrasts comparing conditions being our main outcome. A within-subjects, mixed-effects FLAME 1 model was used to investigate differences in whole-brain activation on placebo and the MC4Ra. Separate models were constructed for scan 1 and scan 2. Statistical images were thresholded using clusters determined by *Z* > 2.3 and a corrected cluster significance threshold at *P* = 0.05.

### Long videos and resting state.

A priori ROIs were defined from a search of the term “sexual” on the meta-analytic website Neurosynth (www.neurosynth.org). This provided data from an automated meta-analysis of 81 studies relating to sexual function. From this, we defined 6 sexual network ROIs: the amygdala, the hypothalamus, the insula, the precentral gyrus, the striatum, and the thalamus (Supplemental Figure 6). The time series from each of these ROIs was extracted from the denoised long video and resting-state scans. Pairwise correlations then compared each ROI using Pearson’s correlations and custom python code. The resulting *r* values were then transformed to *Z* scores using Fisher’s transformation test (91). A 2 × 2 ANOVA was then performed on each region pair to test the effect of the drug (placebo vs. MC4Ra) and the task (long video vs. resting state) as well as the interaction. The *Z* scores were then displayed graphically in a correlation matrix. In the matrices where a difference was shown, post hoc Tukey’s tests were conducted to show significant differences between both the drug and task conditions.

### Statistics.

The statistical analysis plan was designed in collaboration with an independent statistician. Paired *t* tests were performed on the short video and control task fMRI data to assess differences between MC4Ra and placebo in a GLM. These were all cluster corrected to *Z* > 2.3 and *P* = 0.05. For the connectivity analysis, 15, 2 (MC4Ra vs. placebo) by 2 (task, long videos vs. resting) ANOVAs were conducted. Post hoc Tukey’s tests were carried out to investigate the difference between tasks in each drug condition. An α threshold of *P* < 0.05 identified statistical significance except for the connectivity analyses, in which the threshold was reduced to *P* < 0.01 (to adjust for the number of analyses performed). Psychometric data were not normally distributed by D’Agostino-Pearson testing and were therefore analyzed using a Wilcoxon matched-pairs test. Hormone level analysis was performed using mixed-effects models, with *P* < 0.05 considered statistically significant. A McNemar test was conducted on categorical data collected from a 24-hour post-administration sexual desire questionnaire.

### Study approval.

Ethics approval was granted by the London Brent Research Ethics Committee (REC ref: 19/LO/1161), and the study was registered with ClinicalTrials.gov (NCT04179734). Participants provided written informed consent. The study was conducted in accordance with Declaration of Helsinki principles and the International Council for Harmonization Guidelines on Good Clinical Practice.

## Author contributions

WSD, ANC, TH, MBW, NE, DG, and LT conceived and designed the study. LT, EGM, BP, ECA, MP, SS, MM, PCE, BM, TH, NE, and MBW collected the data. LT, TH, ANC, NE, MBW, AA, and PB analyzed the data. LT, TH, EGM, MBW, NE, ANC, and WSD wrote the manuscript. All authors reviewed the final version of the manuscript.

## Supplementary Material

Supplemental data

ICMJE disclosure forms

## Version 1 10/03/2022

Electronic publication

## References

[1] Shruti S, Prevot V (2016). A kiss to set the rhythm. Elife.

[2] Babwah AV (2020). The wonderful and masterful G protein-coupled receptor (GPCR): A focus on signaling mechanisms and the neuroendocrine control of fertility. Mol Cell Endocrinol.

[3] Gresham R (2016). Kisspeptin in the medial amygdala and sexual behavior in male rats. Neurosci Lett.

[4] Adekunbi DA (2018). Kisspeptin neurones in the posterodorsal medial amygdala modulate sexual partner preference and anxiety in male mice. J Neuroendocrinol.

[5] Mills EGA (2021). Functions of galanin, spexin and kisspeptin in metabolism, mood and behaviour. Nat Rev Endocrinol.

[6] Calabrò RS (2019). Neuroanatomy and function of human sexual behavior: a neglected or unknown issue?. Brain Behav.

[7] Parish SJ, Hahn SR (2016). Hypoactive sexual desire disorder: a review of epidemiology, biopsychology, diagnosis, and treatment. Sex Med Rev.

[9] West SL (2008). Prevalence of low sexual desire and hypoactive sexual desire disorder in a nationally representative sample of US women. Arch Intern Med.

[10] Shifren JL (2008). Sexual problems and distress in United States women: prevalence and correlates. Obstet Gynecol.

[11] Goldmeier D (2004). Cost implications of sexual dysfunction: the female picture. Int J Impot Res.

[12] Foley K (2010). Healthcare resource utilization and expenditures of women diagnosed with hypoactive sexual desire disorder. J Med Econ.

[13] Cacioppo S (2017). Neuroimaging of female sexual desire and hypoactive sexual desire disorder. Sex Med Rev.

[14] Cone RD (2005). Anatomy and regulation of the central melanocortin system. Nat Neurosci.

[15] Pfaus JG (2009). Pathways of sexual desire. J Sex Med.

[16] Mountjoy KG (1994). Localization of the melanocortin-4 receptor (MC4-R) in neuroendocrine and autonomic control circuits in the brain. Mol Endocrinol.

[17] Kishi T (2003). Expression of melanocortin 4 receptor mRNA in the central nervous system of the rat. J Comp Neurol.

[18] Molinoff PB (2003). PT-141: a melanocortin agonist for the treatment of sexual dysfunction. Ann N Y Acad Sci.

[19] Clayton AH (2016). Bremelanotide for female sexual dysfunctions in premenopausal women: a randomized, placebo-controlled dose-finding trial. Womens Health (Lond).

[20] Kingsberg SA (2019). Bremelanotide for the treatment of hypoactive sexual desire disorder: two randomized phase 3 trials. Obstet Gynecol.

[21] Dhillon S, Keam SJ (2019). Bremelanotide: first approval. Drugs.

[22] Clément K (2020). Efficacy and safety of setmelanotide, an MC4R agonist, in individuals with severe obesity due to LEPR or POMC deficiency: single-arm, open-label, multicentre, phase 3 trials. Lancet Diabetes Endocrinol.

[23] Sonkusare S (2019). Naturalistic stimuli in neuroscience: critically acclaimed. Trends Cogn Sci.

[24] Huynh HK (2012). High-intensity erotic visual stimuli de-activate the primary visual cortex in women. J Sex Med.

[25] Hutchison RM (2013). Dynamic functional connectivity: promise, issues, and interpretations. Neuroimage.

[26] Voon V (2014). Neural correlates of sexual cue reactivity in individuals with and without compulsive sexual behaviours. PLoS One.

[27] Tao YX (2010). The melanocortin-4 receptor: physiology, pharmacology, and pathophysiology. Endocr Rev.

[28] Mul JD (2012). Melanocortin receptor 4 deficiency affects body weight regulation, grooming behavior, and substrate preference in the rat. Obesity (Silver Spring).

[29] Goldstein I (2017). Hypoactive sexual desire disorder: international society for the study of women’s sexual health (ISSWSH) expert consensus panel review. Mayo Clin Proc.

[30] Clayton AH (2010). The pathophysiology of hypoactive sexual desire disorder in women. Int J Gynaecol Obstet.

[31] Decety J, Cacioppo S (2012). The speed of morality: a high-density electrical neuroimaging study. J Neurophysiol.

[32] Tang A (2016). Processing of different types of social threat in shyness: Preliminary findings of distinct functional neural connectivity. Soc Neurosci.

[33] Ortigue S, Bianchi-Demicheli F (2008). The chronoarchitecture of human sexual desire: a high-density electrical mapping study. Neuroimage.

[34] Clayton AH (2018). Evaluation and management of hypoactive sexual desire disorder. Sex Med.

[35] Baid R, Agarwal R (2018). Flibanserin: a controversial drug for female hypoactive sexual desire disorder. Ind Psychiatry J.

[36] Davis SR (2019). Global consensus position statement on the use of testosterone therapy for women. J Clin Endocrinol Metab.

[37] Bretas RV (2020). Secondary somatosensory cortex of primates: beyond body maps, toward conscious self-in-the-world maps. Exp Brain Res.

[38] Salvato G (2020). Building the bodily self-awareness: evidence for the convergence between interoceptive and exteroceptive information in a multilevel kernel density analysis study. Hum Brain Mapp.

[39] Bancroft J (2009). The dual control model: current status and future directions. J Sex Res.

[40] Stoodley CJ, Schmahmann JD (2009). Functional topography in the human cerebellum: A meta-analysis of neuroimaging studies. Neuroimage.

[41] Beauregard M (2001). Neural correlates of conscious self-regulation of emotion. J Neurosci.

[42] Stoléru S (2012). Functional neuroimaging studies of sexual arousal and orgasm in healthy men and women: a review and meta-analysis. Neurosci Biobehav Rev.

[43] Arnow BA (2009). Women with hypoactive sexual desire disorder compared with normal females: a functional magnetic resonance imaging study. Neuroscience.

[44] Woodard TL (2013). Brain activation patterns in women with acquired hypoactive sexual desire disorder and women with normal sexual function: a cross-sectional pilot study. Fertil Steril.

[45] Wise NJ (2017). Brain activity unique to orgasm in women: an fMRI analysis. J Sex Med.

[46] Georgiadis JR, Kringelbach ML (2012). The human sexual response cycle: brain imaging evidence linking sex to other pleasures. Prog Neurobiol.

[47] Meston CM (2004). Women’s orgasm. Annu Rev Sex Res.

[48] Komisaruk BR, Whipple B (2005). Functional MRI of the brain during orgasm in women. Annu Rev Sex Res.

[49] Kistler-Heer V (2008). Different developmental patterns of melanocortin MC3 and MC4 receptor mRNA: predominance of Mc4 in fetal rat nervous system. J Neuroendocrinol.

[50] Sundaram T (2010). Time-course analysis of the neuroanatomical correlates of sexual arousal evoked by erotic video stimuli in healthy males. Korean J Radiol.

[51] Stoléru S (1999). Neuroanatomical correlates of visually evoked sexual arousal in human males. Arch Sex Behav.

[52] Redouté J (2000). Brain processing of visual sexual stimuli in human males. Hum Brain Mapp.

[53] Moulier V (2006). Neuroanatomical correlates of penile erection evoked by photographic stimuli in human males. Neuroimage.

[54] Stoléru S (2003). Brain processing of visual sexual stimuli in men with hypoactive sexual desire disorder. Psychiatry Res.

[55] Decety J, Grèzes J (1999). Neural mechanisms subserving the perception of human actions. Trends Cogn Sci.

[56] Manfredi-Lozano M (2016). Defining a novel leptin-melanocortin-kisspeptin pathway involved in the metabolic control of puberty. Mol Metab.

[57] Israel DD (2012). Effects of leptin and melanocortin signaling interactions on pubertal development and reproduction. Endocrinology.

[58] Reid RL (1981). Alpha-melanocyte stimulating hormone induces gonadotropin release. J Clin Endocrinol Metab.

[59] Reid RL (1984). Gonadotropin-releasing activity of alpha-melanocyte-stimulating hormone in normal subjects and in subjects with hypothalamic-pituitary dysfunction. J Clin Endocrinol Metab.

[60] Acevedo-Rodriguez A (2018). Emerging insights into hypothalamic-pituitary-gonadal axis regulation and interaction with stress signalling. J Neuroendocrinol.

[61] Goldstat R (2003). Transdermal testosterone therapy improves well-being, mood, and sexual function in premenopausal women. Menopause.

[62] Fooladi E (2014). Testosterone improves antidepressant-emergent loss of libido in women: findings from a randomized, double-blind, placebo-controlled trial. J Sex Med.

[63] Zheng J (2020). Associations between androgens and sexual function in premenopausal women: a cross-sectional study. Lancet Diabetes Endocrinol.

[64] Chung WS (2013). Gender difference in brain activation to audio-visual sexual stimulation; do women and men experience the same level of arousal in response to the same video clip?. Int J Impot Res.

[65] Chapman CD (2018). Experimenter gender and replicability in science. Sci Adv.

[66] Bianchi-Demicheli F (2011). Neural bases of hypoactive sexual desire disorder in women: an event-related FMRI study. J Sex Med.

[67] Nimbi FM (2020). Sexual desire and fantasies in the LGBT+ community: focus on lesbian women and gay men. Curr Sex Heal Reports.

[68] Clément K (2018). MC4R agonism promotes durable weight loss in patients with leptin receptor deficiency. Nat Med.

[69] Rosen RC (2000). The Female Sexual Function Index (FSFI): a multidimensional self-report instrument for the assessment of female sexual function. J Sex Marital Ther.

[70] Derogatis LR (2021). Psychometric validation of the female sexual distress scale-desire/arousal/orgasm. J Patient Rep Outcomes.

[71] Comninos AN (2017). Kisspeptin modulates sexual and emotional brain processing in humans. J Clin Invest.

[72] Murphy K, Garavan H (2004). An empirical investigation into the number of subjects required for an event-related fMRI study. Neuroimage.

[73] Comninos AN (2016). Kisspeptin signaling in the amygdala modulates reproductive hormone secretion. Brain Struct Funct.

[74] Comninos AN, Dhillo WS (2018). Emerging roles of kisspeptin in sexual and emotional brain processing. Neuroendocrinology.

[75] Comninos AN (2018). Modulations of human resting brain connectivity by kisspeptin enhance sexual and emotional functions. JCI Insight.

[76] Yang L (2020). Kisspeptin enhances brain responses to olfactory and visual cues of attraction in men. JCI Insight.

[77] Yang L (2021). The effects of kisspeptin on brain response to food images and psychometric parameters of appetite in healthy men. J Clin Endocrinol Metab.

[78] Comninos AN (2021). Kisspeptin modulates gamma-aminobutyric acid levels in the human brain. Psychoneuroendocrinology.

[79] Mills EG (2022). Current perspectives on kisspeptins role in behaviour. Front Endocrinol (Lausanne).

[80] Shirazi TN (2018). Menstrual cycle phase predicts women’s hormonal responses to sexual stimuli. Horm Behav.

[81] Toledano R, Pfaus J (2006). The Sexual Arousal and Desire Inventory (SADI): a multidimensional scale to assess subjective sexual arousal and desire. J Sex Med.

[82] Aitken RC (1969). Measurement of feelings using visual analogue scales. Proc R Soc Med.

[83] Bates ME, Lemay EP (2004). The d2 Test of attention: construct validity and extensions in scoring techniques. J Int Neuropsychol Soc.

[84] Bourke JH, Wall MB (2015). phMRI: methodological considerations for mitigating potential confounding factors. Front Neurosci.

[85] Harvey JL (2018). A short, robust brain activation control task optimised for pharmacological fMRI studies. PeerJ.

[86] Jenkinson M (2002). Improved optimization for the robust and accurate linear registration and motion correction of brain images. Neuroimage.

[87] https://www.fmrib.ox.ac.uk/datasets/techrep/tr07ja1/tr07ja1.pdf.

[88] https://www.fmrib.ox.ac.uk/datasets/techrep/tr07ja2/tr07ja2.pdf.

[89] Smith SM (2002). Fast robust automated brain extraction. Hum Brain Mapp.

[90] Woolrich MW (2001). Temporal autocorrelation in univariate linear modeling of FMRI data. Neuroimage.

[91] Fisher RA (1915). Frequency distribution of the values of the correlation coefficient in samples from an indefinitely large population. Biometrika.

