# Supplementary material for: Melanocortin 4 receptor agonism enhances sexual brain processing in women with hypoactive sexual desire disorder
Source: J Clin Invest. 2022 Oct 3;132(19):e152341. doi: 10.1172/JCI152341 (PMC9525110; doi:10.1172/JCI152341)
Supplement: Supplemental data [file jci-132-152341-s017.pdf]

Supplemental Data

Supplemental Figure 1: Group average task effects across both melanocortin-4 receptor agonism, placebo conditions and scan times, while viewing erotic and control exercise videos.

Red/yellow areas show group activation to erotic videos, while blue/green areas show deactivation to erotic videos. Significant clusters corrected for multiple comparisons.

$Z = 2.3$, $P < 0.05$, $n = 31$.

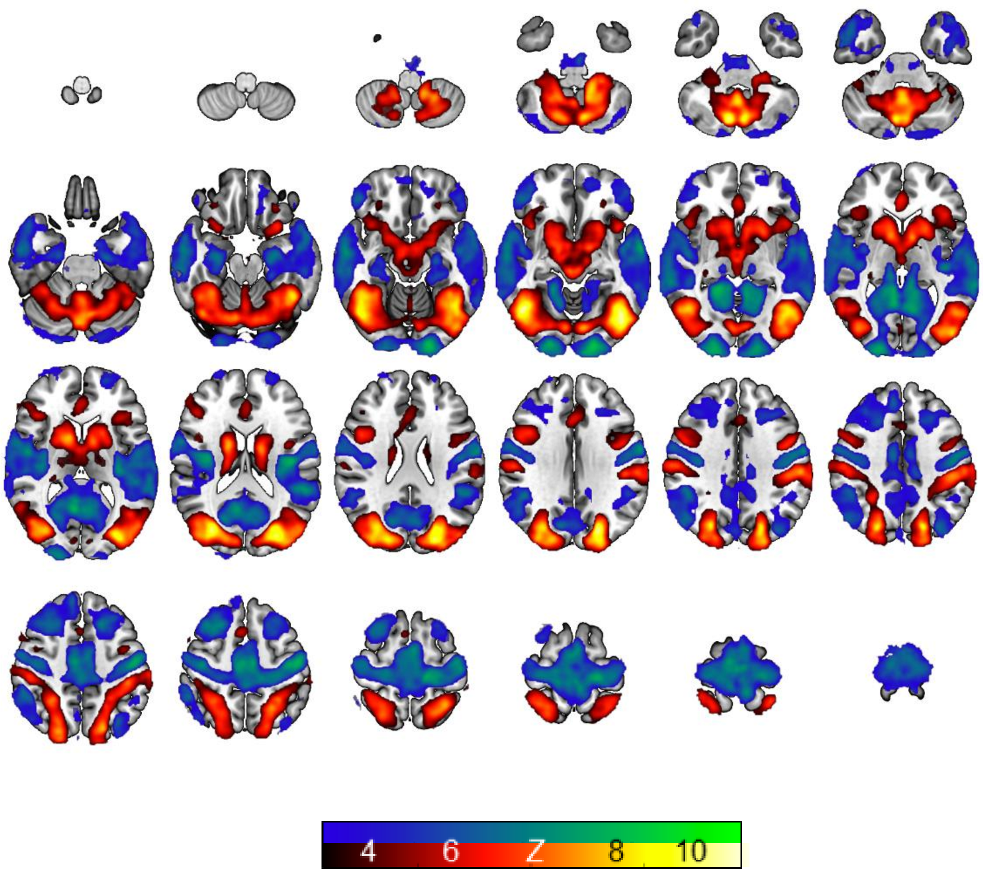

Supplemental Figure 2: Melanocortin-4 receptor (MC4R) agonism has no effect on brain processing of auditory, eye movement, motor, or visual stimuli.

Top row shows the group mean from all scans (MC4Ra and Placebo and Scan 1 and 2) for the auditory, eye movement, motor and visual stimuli from the control task. All conditions performed as expected and produced a well-defined pattern of activation, consistent with the specific stimulus condition. The second row shows the drug comparison MC4Ra > placebo. No drug effects were seen in any condition. Significant clusters are corrected for multiple comparisons. $Z = 2.3$, $P < 0.05$, $n = 31$.

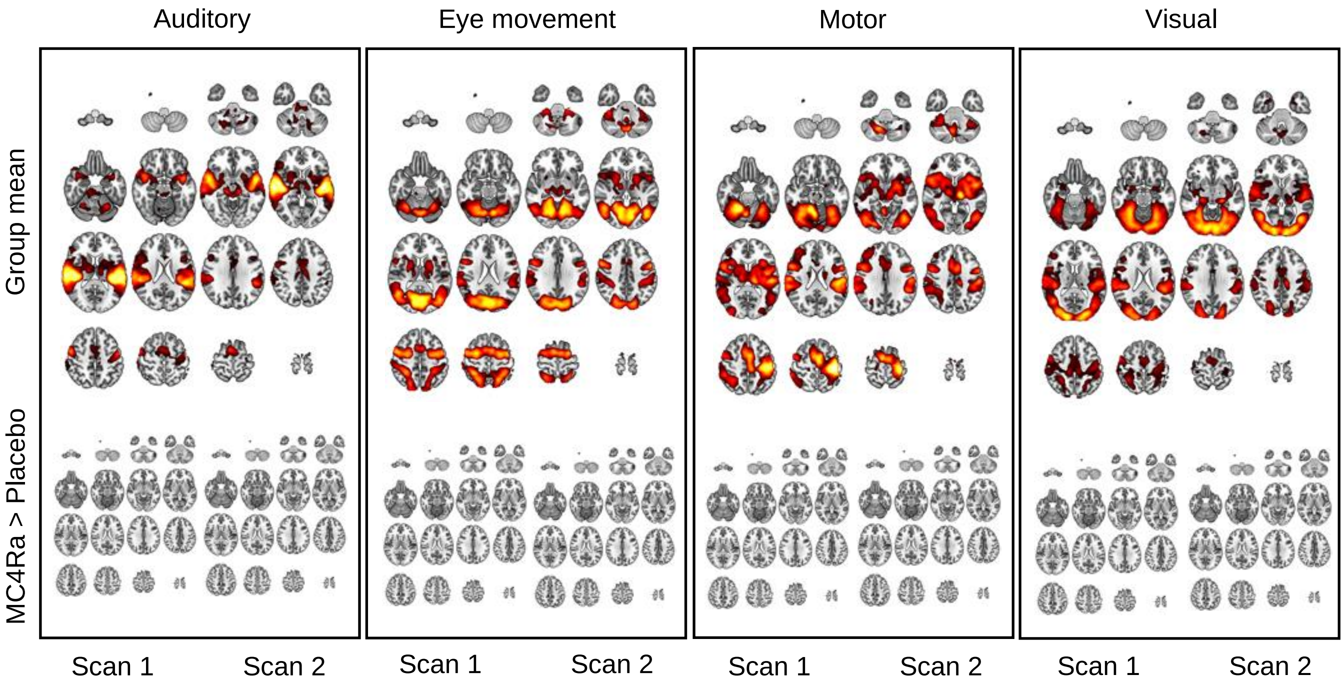

Supplemental Figure 3: Effect of melanocortin-4 receptor (MC4R) agonism on circulating reproductive hormonal levels.

MC4R agonism led to a small increase in LH levels (**A**), FSH levels (**B**), and testosterone (**C**) with no effect on estradiol (**D**) or progesterone (**E**). Data depict mean ± SEM, mixed-effects model.

* $P \leq 0.05$ ** $P \leq 0.01$ *** $P \leq 0.001$, $n = 31$.

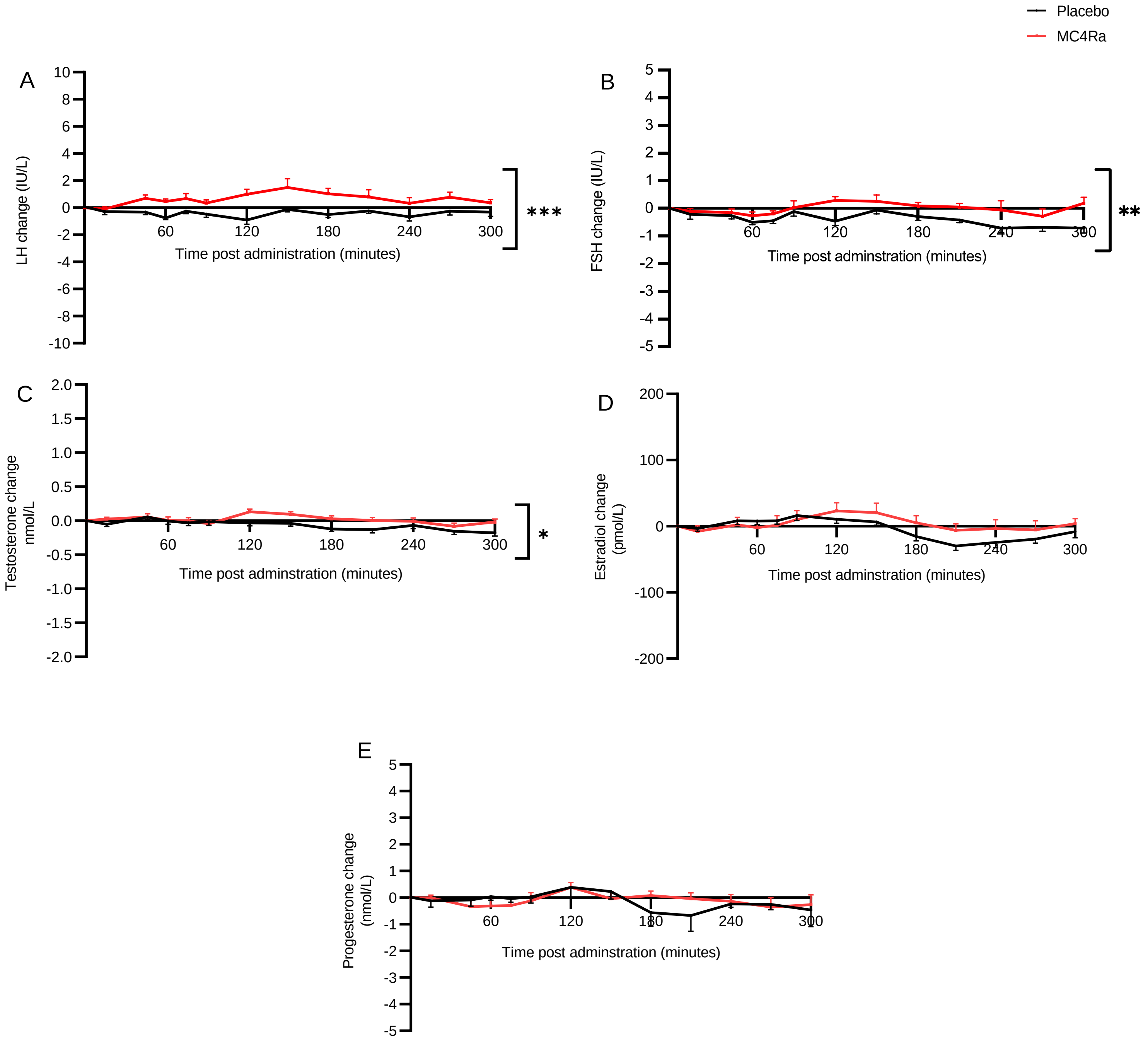

Supplemental Figure 4: Melanocortin-4 receptor (MC4R) agonism significantly increases nausea, fullness and reduced food intake but has no effect on attention.

Data show results from visual analogue scale (VAS) scores. MC4R agonism led to an increase in nausea after Scan 1 (**A**) and Scan 2 (**B**). MC4R agonism led to an increase in fullness after Scan 1 (**C**) and food intake was reduced 3-hours after administration of the MC4R agonist, compared with placebo (**D**). Attention, assessed using the D2 test, was unaltered following MC4R agonism compared to placebo (**E**). Data presented as within-participant paired raw data and median and interquartile range. Wilcoxon matched-pairs test, *** $P \leq 0.001$, $n = 31$.

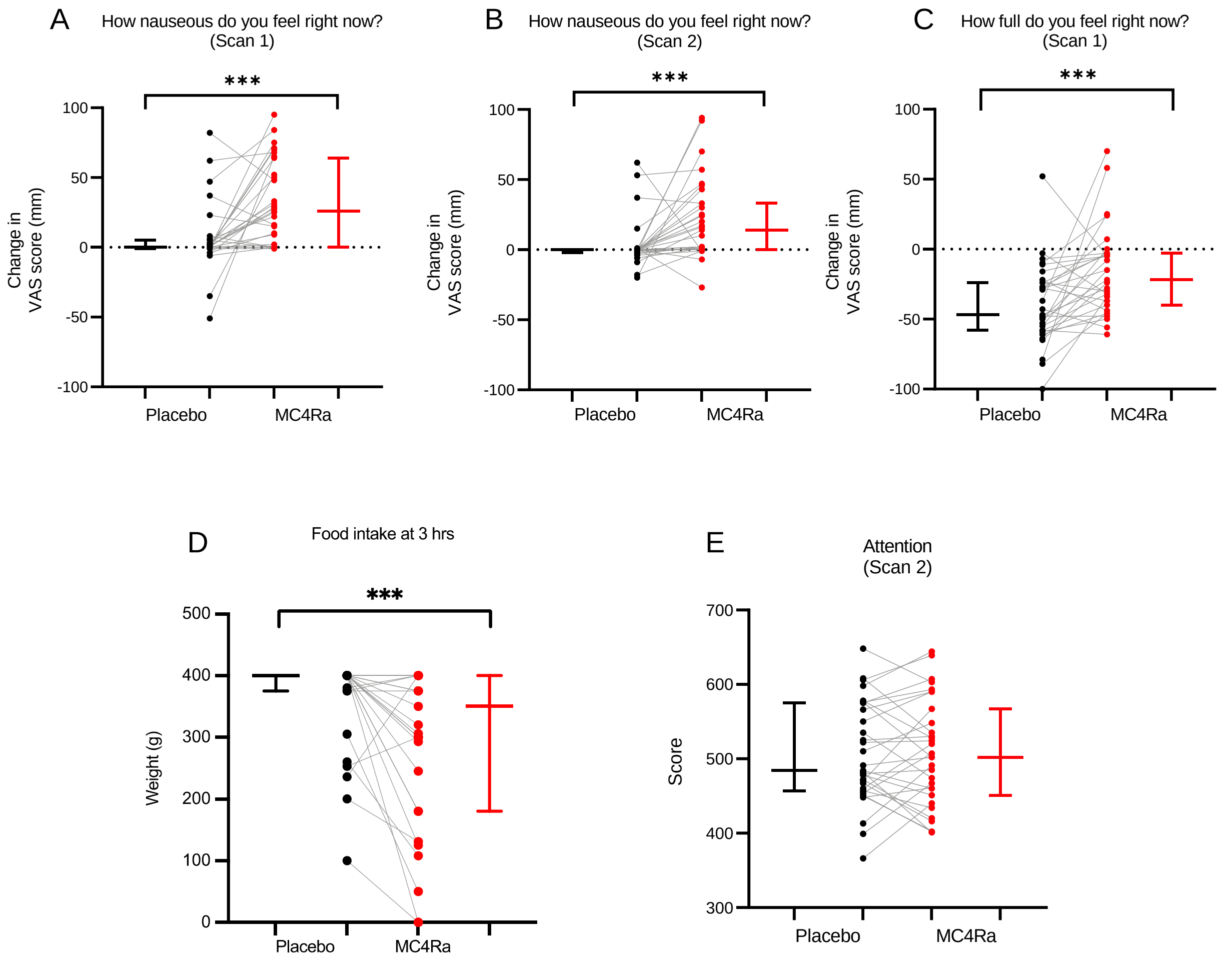

Supplemental Figure 5: Sexual Arousal and Desire Inventory (SADI) scores.

There was no significant change in scores from baseline following melanocortin-4 receptor agonist (MC4R) agonism compared to placebo in the evaluative (P = 0.7131) (**A**), negative (P = 0.7003) (**B**), physiological (P = 0.1008) (**C**), or motivational (P = 0.9672) (**D**) domains. Scan 1 data presented as within-participant paired raw data and median and interquartile range. Similarly, no differences were observed in Scan 2 (data not shown). Wilcoxon matched-pairs test, n = 31.

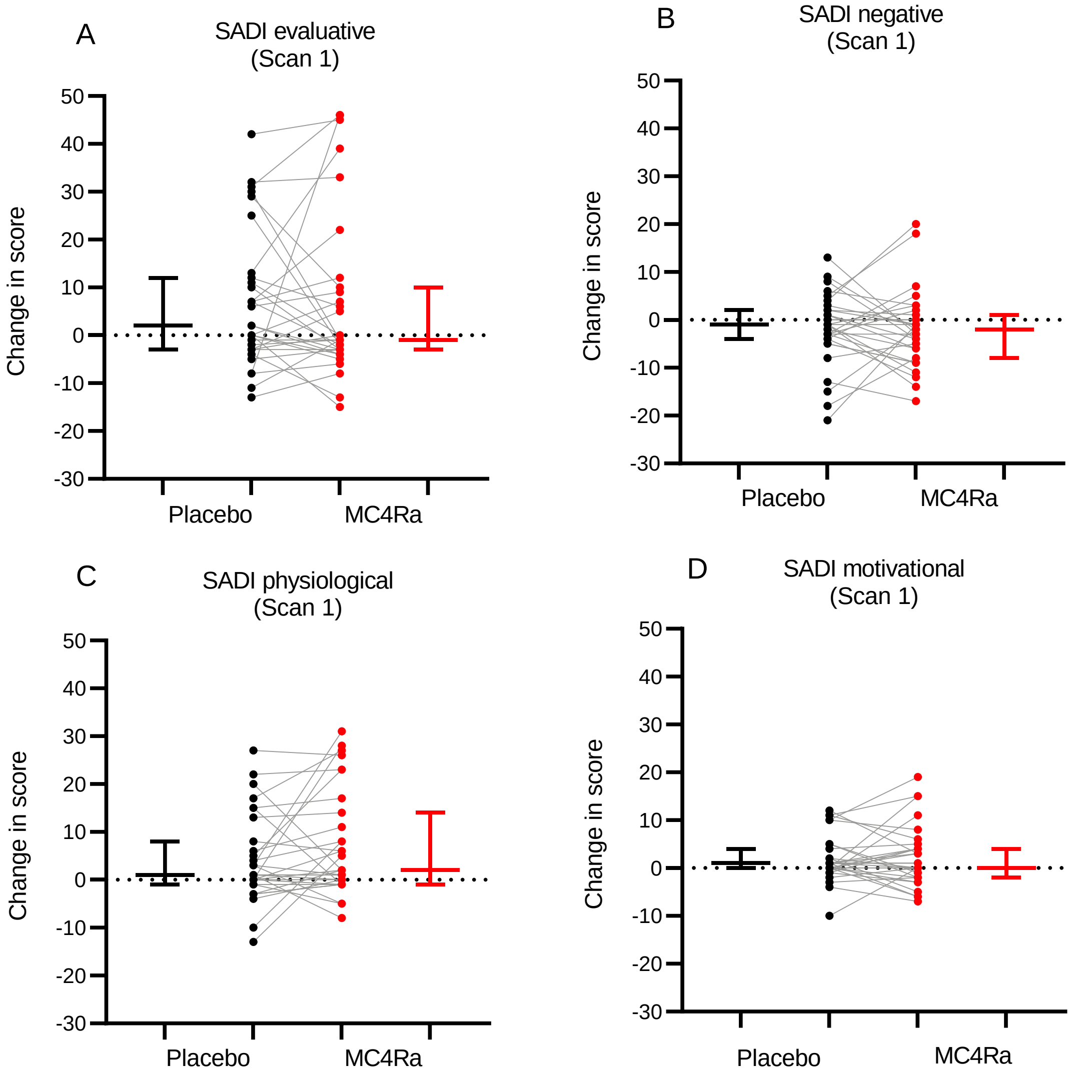

Supplemental Figure 6: Regions of interest (ROI).

A priori ROIs were defined from a search of the term 'sexual' on the metanalytic website Neurosynth [www.neurosynth.org]. This provided data from an automated meta-analysis of 81 studies relating to sexual function. From this, the following six sexual-network ROIs were defined: **Amygdala** **Hypothalamus** **Insula** **Precentral gyrus** **Striatum** **Thalamus**.

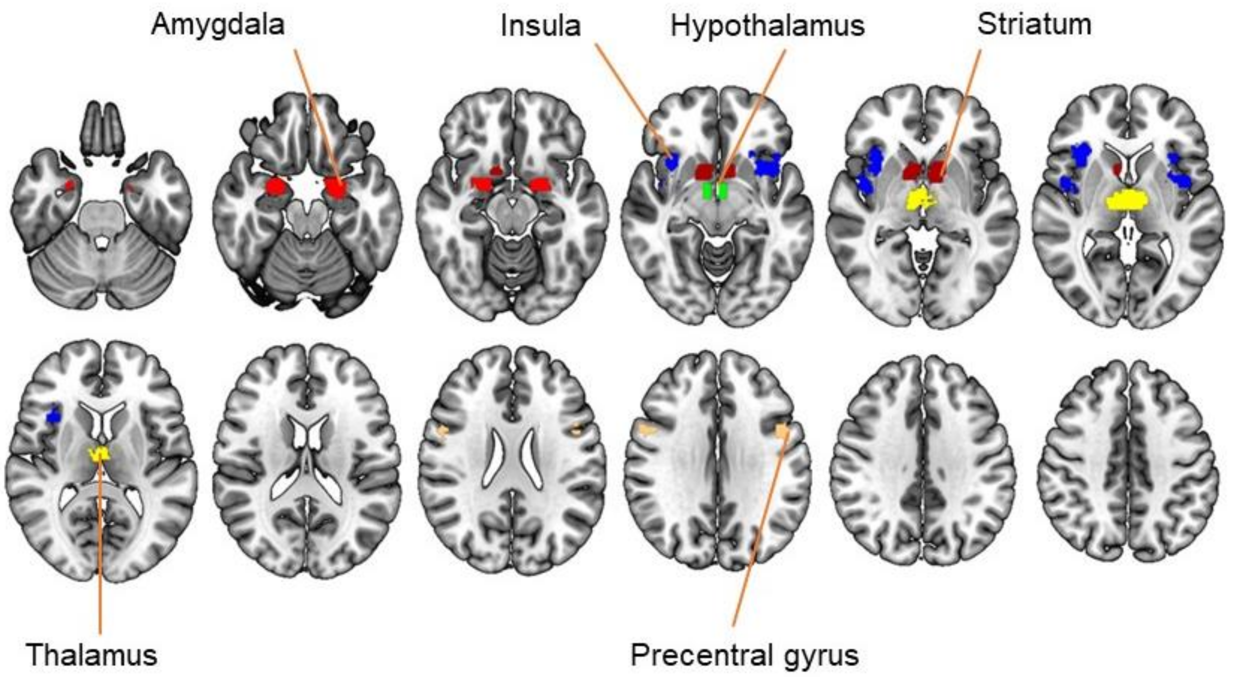

Supplemental Table 1: Baseline characteristics

Characteristic	Median (IQR) or number (%)
**Age (years)**	29 (25, 36)
**BMI (kg/m^2)**	22.4 (20.1, 26.2)
**Ethnicity**	White 18 (58.1)<br>Asian 5 (16.1)<br>Black 3 (9.7)<br>Mixed 3 (9.7)<br>Hispanic 2 (6.5)
**Duration of relationship (months)**	36 (19, 54)
**Age of partner (years)**	31 (29, 41)
**Intercourse (frequency per month)**	2 (1,3)
**Parous**	Yes 7 (22.6)<br>No 24 (77.4)
**Duration of HSDD (months)**	36 (18, 60)
**FSFI total score**	16 (12, 19)
**FSFI desire domain**	1.2 (1.2, 1.8)
**FSDS-DAO total score**	41 (34, 47)
**FSDS-DAO item 13**	4 (3,4)
**PHQ-9**	2 (2,3)
**GAD-7**	1 (0,3)
**LH (IU/L)**	4.8 (3.2,6.6)
**FSH (IU/L)**	3.9 (3.0, 5.2)
**Estradiol (pmol/L)**	379 (236, 443)
**Testosterone (nmol/L)**	1.1 (0.9, 1.3)

GAD-7, Generalised Anxiety Disorder-7; FSDS-DAO, Female Sexual Distress Scale – Desire/ Arousal/ Orgasm; FSFI, Female Sexual Function Index; FSH, follicle-stimulating hormone; HSDD, hypoactive sexual desire disorder; LH, leutinizing hormone; PHQ-9, Patient Health Questionnaire-9. Inclusion criteria included a diagnosis of generalized, acquired HSDD in accordance with DSM-IV-TR criteria, of at least six-month duration, confirmed with a total FSFI score ≤ 26 and a score ≤ 5 in the desire domain, as well as a score of ≥ 18 on the FSDS-DAO assessment tool. The PHQ-9 and GAD-7 questionnaires were performed to exclude depression and anxiety, respectively. Data presented as median and interquarlite range, categorical data presented as number of participants (%), n = 31.

Supplemental Table 2: Clusters with enhanced activation or deactivation by MC4Ra on whole brain analysis

		X	Y	Z	*Voxels*	*Z max*	*P value*
**Scan 1: Erotic > exercise**							
Secondary somatosensory cortex (right)	Deactivation	58.0	-4.16	19.1	691	3.60	0.004
Secondary somatosensory cortex (left)	Deactivation	-59.5	-7.58	27.1	607	3.51	0.01
Cerebellum (right)	Activation	22.7	-51.0	-18.3	517	3.61	0.02
**Scan 2: Erotic > exercise**							
Supplementary motor area	Activation	-0.32	-6.52	67.9	733	4.03	0.002

Data derived from whole brain analysis during the erotic videos and facial attraction task. X, Y and Z are coordinates in a standardized Euclidean space based on the MNI152 brain template and represent the centre of gravity for discrete activation/ deactivation clusters observed in the group-level analyses of treatment effects (MC4Ra vs placebo). **X** = sagittal, **Y** = axial, **Z** = coronal, ***Z* max** = maximum *Z* value of the cluster, *n* = 31.
